# Antithrombotic therapy for hepatic artery thrombosis following liver transplantation: a case report and literature review

**DOI:** 10.3389/fphar.2026.1807440

**Published:** 2026-07-29

**Authors:** Ziwan Guan, Sha Qiu

**Affiliations:** 1 Department of Pharmacy, The First Affiliated Hospital of Jinan University, Guangzhou, China; 2 Department of Pharmacy, The Second Affiliated Hospital of Chongqing Medical University, Chongqing, China

**Keywords:** anticoagulant, antiplatelet, case report, hepatic artery thrombosis, liver transplantation

## Abstract

**Background:**

Hepatic artery thrombosis (HAT) is one of the most severe complications following liver transplantation (LT). Its prevention requires balancing the risks of bleeding and embolism. Due to the incomplete recovery of the coagulation and fibrinolytic system function in LT patients and considerable individual variability, there is currently no standardized protocol for HAT prevention.

**Case Presentation:**

This report presents a case of HAT in a patient with liver failure following LT. Following surgical thrombectomy and hepatic artery resection and anastomosis, the patient received combined antiplatelet and parenteral anticoagulant therapy during hospitalization. At discharge, sequential therapy was transitioned to direct oral anticoagulants (DOACs) for long-term prevention of HAT recurrence, resulting in favorable outcomes. This study also reviews relevant studies on HAT following LT and summarizes the current evidence-based treatment and antithrombotic prophylaxis strategies for HAT.

**Conclusion:**

Antithrombotic strategies vary according to the center. Although antiplatelet therapy has the highest level of evidence for HAT prevention, recent clinical experience also suggests a role for anticoagulants in selected patient populations. Regimen selection requires balancing thrombotic and hemorrhagic risks. In this case, we found that novel biomarkers (e.g., thrombin–antithrombin and plasmin–α2-plasmin inhibitor complexes) may guide anticoagulation decisions, and DOACs represent a promising long-term option for HAT prevention.

## 1 Introduction

Since the first human liver transplantation (LT) in 1963, the procedure has evolved over decades and is now widely applied to patients with end-stage liver disease, primary liver malignancies, and acute liver failure, with favorable long-term outcomes. Current reports indicate a 5-year survival rate of 70%–85% among transplant recipients. Nevertheless, various postoperative complications continue to impact long-term prognosis and quality of life. Hepatic artery complications represent relatively uncommon post-LT events, primarily including hepatic artery thrombosis (HAT), hepatic artery stenosis, and hepatic artery pseudoaneurysm (Cizman and Saad, 2023). Among these, the incidence of HAT in adults ranges from 3% to 12% (Kubihal et al., 2023), making it the most severe vascular complication following LT and one of the primary causes of recipient mortality. Based on the onset time, HAT can be classified into early (<1 month) and late (>1 month) HAT (Oh et al., 2001). Because the blood supply to the biliary tract post-LT entirely relies on the reconstructed hepatic artery, HAT causes biliary ischemia, potentially leading to necrosis or late-stage stenosis (Panaro et al., 2011). Therefore, early restoration of hepatic arterial blood flow after HAT is important for the biliary system. The primary treatments for HAT are surgical procedures and endovascular intervention (Xu et al., 2022). Antithrombotic agents, including antiplatelets and anticoagulants, serve as a preventive strategy to reduce the incidence and recurrence of HAT, playing a vital role in LT postoperative management. However, no randomized controlled trials on antithrombotic prophylaxis of HAT have been conducted to date. Current strategies rely solely on monocenter or regional multicenter experience, with their efficacy and safety remaining controversial. Consequently, there is currently no unified standard or guideline for antithrombotic regimens.

This study aims to review the current research progress in the treatment and drug prevention experiences from various centers based on the analysis of the antithrombotic regimen in a case of HAT following LT, providing reliable evidence-based guidance and practical reference for the management of HAT in LT recipients.

## 2 Case presentation

The patient was a 54-year-old male with a height of 168 cm and a weight of 70 kg. He had a medical history of chronic hepatitis B. His mother also had a history of chronic hepatitis B. Approximately 20 days before admission, the patient developed anorexia without an obvious cause, followed by jaundice of the skin and sclera, accompanied by dark urine and bloody stools. Computerized tomography (CT) revealed cirrhotic liver changes and splenomegaly. Following treatment with hepatoprotection, antiviral therapy, nutritional support, plasma exchange, human albumin supplementation, and diuretics at an external hospital, there was no significant improvement in the patient’s symptoms. Consequently, the patient was transferred to our hospital on 8 July 2025. The admission diagnosis was decompensated cirrhosis with liver failure. Upon admission, the patient received aggressive symptomatic supportive therapy including antiviral treatment, hepatoprotection, anti-infection measures, and correction of coagulation function, along with artificial extracorporeal liver support to improve his condition.

On 1 August 2025, the patient underwent total hepatectomy and allogeneic orthotopic LT. Considering the high risk of postoperative thrombosis, fondaparinux sodium 2.5 mg was initiated on 3 August for thromboprophylaxis. However, due to persistent thrombocytopenia and the concomitant elevated bleeding risk, fondaparinux sodium was discontinued on 4 August, with platelet transfusions and recombinant human thrombopoietin (rhTPO) being administered from 4 to 7 August. Immunosuppressive therapy was initiated on 2 August with tacrolimus 1 mg q12 h in combination with mycophenolate mofetil 0.5 g q12 h. On 6 August, the tacrolimus trough concentration was measured at 2.6 ng/mL, which was below the therapeutic range. Subsequent continuous monitoring of trough concentrations was conducted to adjust the dosage, and the tacrolimus dose was ultimately adjusted to 2 mg q12 h. Daily post-LT Doppler ultrasound examinations of the hepatic artery, portal vein, and inferior vena cava revealed an increased resistance index in the hepatic artery, with no remarkable abnormalities in the portal or inferior vena cava. On 7 August, hepatic artery ultrasound showed no detectable arterial blood flow signals. Subsequent contrast-enhanced CT revealed no visualization of the hepatic artery. On the basis of the medical history and preoperative examinations, HAT was suspected. On the same day, percutaneous hepatic arteriography was performed, followed by catheter-directed thrombolysis and arterial stenting when necessary. A 2.4F microcatheter (Merit) was advanced to the origin of the celiac trunk under the guidance of a microguidewire (Terumo) and a V-18 guidewire (Boston). Repeated attempts to advance it to the distal common hepatic artery via superselective catheterization failed, necessitating the termination of the procedure. On 8 August, exploratory laparotomy, hepatic artery thrombectomy, and partial hepatic artery resection and anastomosis were performed. Following thrombectomy, bilateral vessels were repeatedly flushed with heparin, and blood supply was satisfactorily restored.

Following a multidisciplinary consultation involving clinical pharmacists, vascular surgeons, and hematologists, prophylactic antithrombotic therapy with fondaparinux sodium 5 mg subcutaneous injection (sc) once daily (quaque die, qd) combined with aspirin 100 mg oral administration (per os, po) qd was recommended and implemented. On 11 August, hepatic artery ultrasound showed no thrombus, but D-dimer levels were elevated compared with previous readings. The anticoagulant was adjusted to dalteparin 5,000 International Unit (IU) sc q12 h. Subsequent repeated ultrasounds of the hepatic artery, portal vein, inferior vena cava, and abdomen showed no thrombus. The patient was discharged on 29 August, and the sequential therapy was transitioned to rivaroxaban 15 mg po qd. On 31 October, the antithrombotic regimen was changed to rivaroxaban 10 mg po qd, which remains in use. During follow-up, the patient’s liver function parameters gradually improved, with no thrombus formation and no bleeding events. The antithrombotic medication used during hospitalization and follow-up is summarized in Table 1, laboratory monitoring indicators are detailed in Table 2, and liver vascular color Doppler ultrasound results are presented in Table 3.

**TABLE 1:** Antithrombotic drug application of the patient during hospitalization and follow-up periods.

​	After surgical thrombectomy						Discharge				
Date	8.8	8.11	8.13	8.18	8.22	8.27	8.29	9.12	10.3	10.31	Up to now
Aspirin	100 mg po qd						​				
Fondaparinux	5 mg sc qd	​									
Dalteparin	​	5,000 IU sc q12 h					​				
Rivaroxaban	​						15 mg po qd			10 mg po qd	

po, oral administration; qd, once daily; IU, international unit; sc, subcutaneous injection.

**TABLE 2:** Bleeding tendency, coagulation profile, and hepatic–renal function indicators in the patient during hospitalization and follow-up periods.

Date	8.2*	8.3	8.4	8.5	8.6	8.7	8.8#	8.11	8.13	8.18	8.22	8.27	9.12	10.3	10.31	11.13
HGB (g/L)	82	81	71	68	69	71	93	79	82	83	95	95	124	125	139	141
PLT (10^9^/L)	51	49	23	25	58	61	63	105	208	193	366	348	300	188	216	205
D-dimer (ng/mL)	1,553.7	2,694.0	2,239.3	2,541.9	4,963.6	3,832.0	2,888.2	3,127.4	2,895.4	1,905.3	1,435.3	1,321.2	962.7	340.7	-	-
FDP (μg/mL)	12.69	22.21	16.67	17.87	36.11	25.32	20.21	25.37	23.62	15.07	12.69	8.97	-	3.22	-	-
PT (s)	19.6	17.2	15.9	15.3	15.9	16.7	16.2	15.1	13.7	13.0	12.5	13.1	15.9	12.5	-	11.5
INR	1.64	1.39	1.25	1.19	1.25	1.33	1.29	1.19	1.03	0.96	0.91	0.97	1.25	0.92	-	0.82
APTT (s)	52.0	39.8	39.8	37.9	36.1	38.6	43.6	49.5	40.3	41.2	35.8	32.3	37.7	36.1	-	33.6
TT (s)	15.2	15.0	15.9	17.1	16.6	17.0	18.1	13.8	15.0	15.1	15.8	16.7	16.3	16.8	-	18.2
FIB (g/L)	1.67	1.83	1.60	1.38	1.29	0.91	1.13	3.7	3.68	3.26	3.95	2.92	3.41	3.86	-	3.61
AT-III activity (%)	32	45	69	63	74	58	55	48	63	71	95	94	-	112	-	-
TAT (ng/mL)	-	-	-	-	-	-	-	9.76	-	-	-	8.98	-	-	-	-
PIC (μg/mL)	-	-	-	-	-	-	-	1.55	-	-	-	1.62	-	-	-	-
ALB (g/L)	43.7	34.7	37.1	35.3	35.6	31.0	29.0	30.6	31.0	33.9	41.1	39.6	43.5	38.8	44.6	42.1
TBIL (μmol/L)	81.5	54.8	52.2	49.7	41.7	32.9	34.9	39.1	61.3	44.5	38.6	25.2	17.8	12.7	12.5	11.2
ALT (U/L)	134	107	95	85	57	41	46	149	231	70	46	23	12	63	63	58
AST (U/L)	144	67	48	40	19	20	35	176	217	60	46	18	18	48	58	54
sCr (μmol/L)	56.3	44.2	43.4	45.1	43.7	-	45.5	29.7	55.6	83.1	87.5	73.8	78.8	-	96.7	90.7

* Day 1 after liver transplantation; # Day 1 after surgical thrombectomy;

HGB, hemoglobin; PLT, platelet; FDP, fibrin degradation product; PT, prothrombin time; INR, international normalized ratio; APTT, activated partial thromboplastin time; TT, thrombin time; FIB, fibrinogen; AT-III, antithrombin III; TAT, thrombin–antithrombin complex; PIC, plasmin–α2-plasmin inhibitor complex; ALB, albumin; TBIL, total bilirubin; ALT, alanine aminotransferase; AST, aspartate aminotransferase; sCr, serum creatinine.

**TABLE 3:** Color Doppler ultrasound of the hepatic vascular system during hospitalization and follow-up periods.

Date	8.2*	8.3	8.4	8.5	8.6	8.7	8.8#	8.11	8.13	8.18	8.22	8.28	9.5	9.26
Hepatic artery	Increased RI					HAT	No obvious abnormalities							
Portal vein	No obvious abnormalities													
Inferior vena cava	No obvious abnormalities													

*Day 1 after liver transplantation; # Day 1 after surgical thrombectomy;

RI, resistance index; HAT, hepatic artery thrombosis.

## 3 Discussion

### 3.1 Risk factors for HAT following LT

The peak incidence of HAT occurs within the first 2 weeks postoperatively. The risk factors for HAT induction include surgical technical factors [e.g., anastomotic technique] (Nemes et al., 2013; Herrero et al., 2017) and prolonged warm–cold ischemia times) and vascular [e.g., anatomical anomalies (Warner et al., 2011), donor–recipient vessel size mismatch (Bastón Castiñeiras et al., 2022), and vascular condition (Mourad et al., 2014)], and postoperative patient factors [e.g., hypercoagulable state, rejection, infection (Unal et al., 2013), and massive transfusion (Miyagi et al., 2013; Xue et al., 2018)] factors. HAT is currently considered a multifactorial outcome. Additionally, the postoperative new liver synthesizes procoagulant factors more rapidly and earlier, but produces anticoagulant substances at a slower and delayed rate (Ozier et al., 2001). This means that the restoration sequence and progression of procoagulant, anticoagulant, and fibrinolytic mechanisms are not fully coordinated. This asynchronous recovery of coagulation function, combined with factors such as donor liver quality, the extent of ischemia and reperfusion injury, blood flow in the transplanted liver, medications, rejection reactions, and infection, collectively leads to the formation of a hypercoagulable state near the vascular anastomosis site, resulting in HAT development.

In this case, the primary risk factors for HAT development included the hepatic artery anastomosis technique (end-to-end anastomosis using 7-0 Prolene sutures, with an anastomosis duration of 99 minutes), postoperative vasoconstrictor use (norepinephrine administered at 1–2 August for a total of 16 mg), which caused vascular endothelial injury and abnormal blood flow, and administration of platelets, rhTPO, fresh frozen plasma, concentrated red blood cells, and other medications that may have induced an acquired hypercoagulable state. Postoperative rejection reactions or suboptimal immunosuppressant concentrations can result in vascular rejection reactions that directly damage the vascular endothelium and induce thrombosis. Conversely, tacrolimus may promote leukocyte–endothelial interactions and microthrombosis by upregulating endothelial adhesion molecules, such as vascular cell adhesion molecule-1 (VCAM-1) and intercellular adhesion molecule-1, and may exacerbate systemic hypercoagulability via platelet activation and the induction of renal vasculopathy. Furthermore, perioperative abdominal infections and the extensive use of glucocorticoids, such as methylprednisolone, have been identified as additional risk factors for thrombosis.

### 3.2 HAT treatment following LT

Prompt detection of HAT formation followed by endovascular urokinase thrombolysis can, if successful, avoid the need for repeat hepatic artery angioplasty (Kogut et al., 2015). This remarkably reduces the incidence of severe postoperative complications caused by HAT and improves the survival rates of the recipients. Patients who exceed the time window or experience thrombolysis failure may face the risk of reoperation, specifically thrombectomy and hepatic artery reconstruction. Undergoing two surgeries within a short period carries substantial risks, and the risk of recurrent thrombosis after thrombectomy and reconstruction is extremely high.

In this case, the patient underwent failed endovascular thrombolysis on 7 August. On 8 August, successful hepatic artery thrombectomy and partial hepatic artery resection and anastomosis were performed. Postoperative Doppler ultrasound confirmed hepatic artery patency with no thrombus formation.

### 3.3 Prevention of recurrent HAT following LT

Pharmacological HAT prevention strategies vary globally across centers. No unified standard exists for the drug selection, dosage, or duration of antithrombotic agents. Primary recent studies on HAT antithrombotic regimens are summarized in Table 4. The literature search strategy is as follows: PubMed, Medline, Web of Science, and Chinese National Knowledge Infrastructure (CNKI) search terms (“hepatic artery thrombosis,” “liver transplantation,” “antithrombotic,” “direct oral anticoagulant,” “aspirin,” “warfarin,” “LMWH,” “anticoagulant,” and “antiplatelet”), date range (2000–2025), language restriction (English or Chinese), and inclusion and exclusion criteria (adult LT recipients, HAT as a primary outcome, and reported antithrombotic intervention).

**TABLE 4:** Recent studies on antithrombotic regimens for HAT.

Author	Year	Country	Organization or data source	Study type	Study size	Treatment of HAT	Antithrombotic drug	Outcome
Craig-Schapiro	2021	United States	Lurie Children’s Hospital of Chicago	Case report	1	Surgical thrombectomy and retransplantation	Hospitalization: heparin and bivalirudin; discharge: enoxaparin	Normal graft function and patent hepatic vasculature on 1-year posttransplant imaging
Zhang	2021	China	The First Affiliated Hospital of Xi′an Jiaotong University	Monocentric series	11	Endovascular thrombolysis	LMWH combined with aspirin/clopidogrel/tirofiban	Nine patients survived, with overall survival rates of 75% at 1, 3, and 5 years; two patients died
Oberkofler	2022	Switzerland	Aspirin4OLT study	Multicentric retrospective cohort study	2,366	No data	Aspirin 75–100 mg/day	The aspirin group had a higher rate of arterial patency and survival
Xu	2022	China	Tianjin First Central Hospital	Monocentric retrospective cohort study	110	Anticoagulant treatment: 19, arterial reconstruction: 1, retransplantation: 2	Heparin/warfarin	94.7% (18/19) of recipients achieved hepatic artery recanalization (4/18) or collateral formation (14/18) after anticoagulant therapy
Feldman	2023	USA	Pediatric Health Information System® (PHIS) database	Multicentric retrospective cohort study	3,259	No data	Anticoagulant citrate dextrose/antithrombin/aspirin/dipyridamole/enoxaparin/heparin	Aspirin was associated with a considerable reduction in the incidence of HAT (*p* < 0.001)
Fennessy	2024	Australia	Children’s Hospital at Westmead	Monocentric retrospective cohort study	282	No data	Vitamin K, antithrombin 3, fresh frozen plasma, heparin, and aspirin	Decreased HAT rates (*p* < 0.001) without an associated increase in bleeding rates was found
Minciuna	2024	The Netherlands	Erasmus MC Transplant Institute	Monocentric retrospective cohort study	836	Arterial reconstruction: 84, arterial anastomosis redo: 18, arterial conduit: 5, thrombectomy: 13	LWMH during the ICU stay. Aspirin/carbasalate calcium for 3 months	In selected high risk patients, aspirin was associated with an 8-fold reduced risk of early HAT and remarkably improved graft survival
Li	2025	China	The First Affiliated Hospital of Xi’an Jiaotong University	Monocentric series	22	Endovascular interventional therapy: 17, surgical thrombectomy: 5	AP: aspirin/tirofiban/clopidogrel; LWMH: enoxaparin; DOACs: rivaroxaban	In total, 16 demonstrated patent hepatic artery flow, 1 awaited retransplantation due to failed arterial thrombolysis, and 2 experienced thrombus recurrence

HAT, hepatic artery thrombosis; AP, antiplatelet; LMWH, low-molecular-weight heparin; DOACs, direct oral anticoagulants.

#### 3.3.1 Antiplatelet therapy

Arterial and venous thromboses exhibit considerable differences in their formation mechanisms, common sites, symptomatic manifestations, and treatment approaches. Arterial thrombosis primarily results from platelet aggregation and frequently occurs in coronary arteries, cerebral arteries, etc. Treatment primarily involves antiplatelet agents or thrombolysis and endovascular intervention. Although HAT formation differs from atherosclerotic thrombosis—primarily arising from surgical factors and donor–recipient vascular factors—previous studies indicate that among LT recipients and patients at higher risk for HAT (Oberkofler et al., 2022), the antiplatelet agent aspirin is the only thromboprophylactic method proven to reduce HAT incidence (Kirchner et al., 2022; Minciuna et al., 2024).

#### 3.3.2 Anticoagulant therapy

Studies have reported that anticoagulants used to prevent venous thrombosis, such as heparin and oral anticoagulants, have not been proven effective or safe for preventing arterial thrombosis, particularly early HAT (Algarni, 2015). Paradoxically, patients undergoing LT face concurrently elevated risks of both thrombosis and bleeding. The literature indicates that even without anticoagulant therapy, the incidence and mortality rates of bleeding complications post-LT remain high. This indicates that anticoagulants may not be suitable for routine use in preventing venous thromboembolism (VTE). Patients concurrently at risk for venous thrombosis such as portal vein thrombosis (PVT) or deep vein thrombosis (DVT) may cautiously consider anticoagulant therapy, but initiation is typically deferred until the international normalized ratio is below 1.5–2.0 and the platelet count exceeds 25–50 × 10^9^/L (Mukerji et al., 2014). Regarding arterial thrombosis risk, recent studies indicate that heparin, low-molecular-weight heparin (LMWH) and bivalirudin can reduce the incidence of HAT (Voulgarelis et al., 2018; Craig-Schapiro et al., 2021; Feldman et al., 2024). Another study reported that 94.7% of recipients undergoing anticoagulation therapy for HAT after heparin or warfarin administration achieved hepatic artery recanalization or collateral circulation formation within 2 weeks, with Doppler ultrasound monitoring showing adequate hepatic blood supply (Xu et al., 2022).

To date, only one case of a patient with COVID-19 with the rare concomitant occurrence of HAT treated solely with direct oral anticoagulants (DOACs) after discharge has been reported (Antunes De Brito et al., 2021). During hospitalization, the patient received LMWH (enoxaparin 60 mg q12 h), and after discharge, rivaroxaban (exact dosage unknown) was administered for 4 months, resulting in thrombus resolution.

#### 3.3.3 Antiplatelet therapy combined with anticoagulant therapy

In a monocenter case series from China, patients with HAT underwent either endovascular thrombolysis or surgical thrombectomy. Postprocedural antithrombotic therapy for endovascular thrombolysis recipients comprised LMWH (enoxaparin 6,000 IU sc q12 h) combined with an antiplatelet agent (tirofiban hydrochloride 5 mg IV continuous infusion q24 h). Following discharge, oral medication therapy, including DOACs (rivaroxaban 20 mg po qd) and an antiplatelet agent (aspirin 100 mg po qd), was administered for 6 months. Patients undergoing surgical thrombectomy and arterial reanastomosis received LMWH (enoxaparin 6,000 IU sc qd) for 1 week alongside an antiplatelet agent (clopidogrel 75 mg po qd) for 6 months. Among the 19 HAT recipients in this study, a median follow-up of 22 months (range 5 - 52) of regular follow-up postdischarge was achieved. Of these, 16 demonstrated patent hepatic artery flow, 1 awaited retransplantation due to failed arterial thrombolysis, and 2 experienced thrombus recurrence (Zhang et al., 2021; Li et al., 2025).

Additionally, in pediatric LT, a novel antithrombotic regimen combining vitamin K, antithrombin III, fresh frozen plasma, heparin, and aspirin has been proposed to maintain effective antithrombotic therapy while reducing bleeding risk (Fennessy et al., 2024).

#### 3.3.4 Antithrombotic regimen in this case

In this case, the patient presented with multiple risk factors for HAT formation, indicating a high risk of recurrence. However, concurrent hepatic coagulation dysfunction and thrombocytopenia posed significant bleeding risks. Thus, balancing antithrombotic therapy to manage both coagulation and fibrinolytic system function represents a therapeutic challenge. Following a multidisciplinary assessment of the patient’s thrombotic and hemorrhagic risks, a decision was made to initiate prophylactic antithrombotic therapy for HAT, PVT, and DVT.

Fondaparinux sodium 2.5 mg sc qd was initiated on 3 August for thromboprophylaxis because of the high risk of postoperative thrombosis. However, due to critically low platelet counts, anticoagulation was temporarily withheld, and instead platelet transfusion was administered, which undoubtedly increased the risk of thrombosis. Following surgical thrombectomy, a multidisciplinary team, including clinical pharmacists, vascular surgeons, and hematologists, collaborated to formulate the antithrombotic regimen. On the basis of existing research, aspirin demonstrated stronger evidence for preventing HAT. The patient exhibited elevated D-dimer levels, and the ratio of thrombin–antithrombin complex (TAT) to plasmin–α2-plasmin inhibitor complex (PIC) (TAT–PIC = 6.30) exceeded 5, indicating hypercoagulability with increased thrombus formation potential (Huang et al., 2024). Additionally, multiple risk factors for thrombosis were present, including peritoneal infection, two major surgeries in a short period, and severe liver disease. Therefore, a combined regimen of aspirin and dalteparin was administered during hospitalization. Dalteparin is primarily renally eliminated with minimal hepatic metabolism, making its pharmacokinetics largely independent of fluctuating hepatic function or cholestasis in the early posttransplant period. This renal clearance confers more predictable plasma concentrations than hepatically metabolized agents, supported by its high subcutaneous bioavailability (∼90%) and manageable half-life (2–4 h). Aspirin is rapidly absorbed and hepatically hydrolyzed, exerting irreversible antiplatelet effects via cyclooxygenase-1 inhibition for the platelet lifespan. Together, these agents provide dual-pathway prophylaxis against HAT through concurrent anticoagulation and antiplatelet mechanisms.

At the time of discharge, the patient exhibited signs of hepatic and coagulative function recovery, as indicated by relevant biochemical markers. However, the persistently elevated D-dimer level, the TAT/PIC ratio, which remained indicative of hypercoagulability, and the incomplete recovery of transplanted liver function indicated that the patient remained in a hypercoagulable state, with an increased risk of late HAT, PVT, and DVT. Therefore, long-term anticoagulation therapy was necessary to prevent thrombus formation after discharge. Referring to a case report (Antunes De Brito et al., 2021) on anticoagulation therapy for patients with COVID-19 with HAT and considering the patient’s medication adherence, rivaroxaban 15 mg po qd was prescribed as monotherapy upon discharge. Notably, the pathophysiological milieu of COVID-19-associated HAT described in that case report (an inflammatory and coagulopathic state in a non-transplant patient) differs fundamentally from post-liver-transplant HAT, which arises from vascular surgical trauma, immunosuppression, and hypercoagulability secondary to graft dysfunction. We cite this case as preliminary evidence supporting the feasibility of DOAC administration in HAT, rather than as a definitive proof of efficacy or safety in LT. Rivaroxaban exhibits a bioavailability of approximately 80%, a half-life of 5–9 hours and generally no requirement for routine monitoring, thereby offering superior outpatient adherence. Approximately two-thirds are metabolized by hepatic CYP3A4/2J2, and one-third is renally excreted. As rivaroxaban is a dual substrate of CYP3A4 and P-glycoprotein, concomitant tacrolimus (a potent dual inhibitor) could theoretically elevate its plasma concentrations. Therefore, periodic monitoring of hepatic function, coagulation parameters, and tacrolimus plasma concentrations remains warranted following hospital discharge.

### 3.4 Patient follow-up and outcome

The patient underwent regular outpatient follow-up. The D-dimer levels gradually decreased and returned to normal one month after discharge. Two months postdischarge, the rivaroxaban dosage was adjusted from 15 to 10 mg po qd. The patient is fully informed and trusts the anticoagulation therapy, with good medication adherence and ongoing follow-up. Imaging studies, including color Doppler ultrasound of the hepatic artery, portal vein, and inferior vena cava, along with laboratory parameters such as blood counts and coagulation profiles, showed no remarkable abnormalities. No bleeding events have occurred. Serial tacrolimus trough concentrations remained within the therapeutic range on repeated monitoring, and no clinically important evidence of a rivaroxaban–tacrolimus interaction was observed in this patient.

### 3.5 Limitations

This study represents only a single case report from our center, and the literature reviewed here largely consists of monocenter experiences or case reports. The primary objective of this case report is hypothesis-generating—to share our institutional experience and provide a clinical scenario for future prospective studies. Furthermore, our findings apply exclusively to secondary prevention in a high risk postoperative setting, with no inferences drawn regarding primary prophylaxis. Future research requires more extensive and larger-scale clinical studies to provide evidence for the primary and secondary prophylaxes of HAT.

Due to the paucity of data on DOAC use following LT, DOAC administration necessitates rigorous postoperative surveillance and close clinical follow-up to ensure both therapeutic efficacy and early detection of complications. In addition to routine monitoring, genetic polymorphisms of Factor X may theoretically influence anticoagulant pharmacodynamics, and donor liver-derived Factor X synthetic capacity represents an underexplored variable in precision medicine for LT recipients. However, our center is currently unable to perform testing for Factor X gene variants. Pharmacogenomics offers a promising research direction for the future development of antithrombotic strategies.

## 4 Conclusion

Given the low incidence of HAT following LT, previous research on its antithrombotic prophylaxis has been limited. Based on a single case analysis at our center, this study presents a review of relevant literature from recent years. We summarized the following insights: ① HAT formation following LT has severe consequences and requires early intervention upon detection, ② surgical treatment and endovascular intervention are first-line treatment options, ③ posttreatment antithrombotic prophylaxis should be adapted in according to the patient’s individual risk factors, and ④ antithrombotic regimens vary across centers. Antiplatelet therapy, especially aspirin, has the highest level of evidence for the prevention of HAT, yet recent clinical experience also suggests a role for anticoagulants in selected patient populations. The formulation of antithrombotic regimens requires comprehensive consideration of the patient’s thrombotic and hemorrhagic risks. ⑤ Novel thrombotic biomarkers (e.g., TAT and PIC) may serve as supplementary indicators to assist in clinical anticoagulation strategy formulation, and ⑥ DOACs represent a potential long-term anticoagulant option for HAT prevention, but their suitability for specific patients, particularly those with multiple prothrombotic risk factors, requires further research.

## Data Availability

The original contributions presented in this study are included in the article/supplementary material; further inquiries can be directed to the corresponding author.
